# Iron-mediated oxidative stress induces PD-L1 expression via activation of c-Myc in lung adenocarcinoma

**DOI:** 10.3389/fcell.2023.1208485

**Published:** 2023-06-12

**Authors:** Anna Martina Battaglia, Alessandro Sacco, Ilenia Aversa, Gianluca Santamaria, Camillo Palmieri, Cirino Botta, Roberto De Stefano, Maurizio Bitetto, Lavinia Petriaggi, Emanuele Giorgio, Concetta Maria Faniello, Francesco Costanzo, Flavia Biamonte

**Affiliations:** ^1^ Laboratory of Biochemistry and Cellular Biology, Department of Experimental and Clinical Medicine, Magna Graecia University of Catanzaro, Catanzaro, Italy; ^2^ Laboratory of Immunology, Department of Experimental and Clinical Medicine, Magna Graecia University of Catanzaro, Catanzaro, Italy; ^3^ Laboratory of Molecular Oncology, Department of Experimental and Clinical Medicine, Magna Graecia University of Catanzaro, Catanzaro, Italy; ^4^ Department of Health Promotion, Mother, and Child Care, Internal Medicine and Medical Specialties, University of Palermo, Palermo, Italy; ^5^ Operational Unit of Anatomic Pathology, Annunziata Hospital, Cosenza, Italy; ^6^ Operational Unit of Thoracic Surgery, Annunziata Hospital, Cosenza, Italy; ^7^ Department of Experimental and Clinical Medicine, Center of Interdepartmental Services (CIS), Magna Graecia University of Catanzaro, Catanzaro, Italy

**Keywords:** iron, PD-L1, TME, lung adenocarcinoma, oxidative stress, c-Myc

## Abstract

**Introduction:** The PD-1/PD-L1 axis is hijacked by lung adenocarcinoma (LUAD) cells to escape immune surveillance. PD-L1 expression in LUAD is affected, among others, by the metabolic trafficking between tumor cells and the tumor microenvironment (TME).

**Methods:** Correlation between PD-L1 expression and iron content within the TME was established on FFPE LUAD tissue samples. The effects of an iron rich microenvironment on PD-L1 mRNA and protein levels were assessed *in vitro* in H460 and A549 LUAD by using qPCR, western blot and flow citometry. c-Myc knockdown was performed to validate the role of this transcription factor on PD-L1 expression. The effects of iron-induced PD-L1 on T cell immune function was assessed by quantifying IFN-γ release in a co-colture system. TCGA dataset was used to analyse the correlation between PD-L1 and CD71 mRNA expression in LUAD patients.

**Results:** In this study, we highlight a significant correlation between iron density within the TME and PD-L1 expression in 16 LUAD tissue specimens. In agreement, we show that a more pronounced innate iron-addicted phenotype, indicated by a higher transferrin receptor CD71 levels, significantly correlates with higher PD-L1 mRNA expression levels in LUAD dataset obtained from TCGA database. *In vitro*, we demonstrate that the addition of Fe^3+^ within the culture media promotes the significant overexpression of PD-L1 in A549 and H460 LUAD cells, through the modulation of its gene transcription mediated by c-Myc. The effects of iron lean on its redox activity since PD-L1 up-regulation is counteracted by treatment with the antioxidant compound trolox. When LUAD cells are co-cultured with CD3/CD28-stimulated T cells in an iron-rich culture condition, PD-L1 up-regulation causes the inhibition of T-lymphocytes activity, as demonstrated by the significant reduction of IFN-γ release.

**Discussion:** Overall, in this study we demonstrate that iron abundance within the TME may enhance PD-L1 expression in LUAD and, thus, open the way for the identification of possible combinatorial strategies that take into account the iron levels within the TME to improve the outcomes of LUAD patients treated with anti-PD-1/PD-L1-based therapies.

## 1 Introduction

In the tumor microenvironment (TME), the programmed cell death protein 1 (PD-1) and its ligand (PD-L1) axis is hijacked by cancer cells to escape immune surveillance. Indeed, PD-L1 on cancer cells binds to tumor-infiltrating lymphocytes (TILs) and impairs their activation, through the inhibition of their proliferation, survival, and effector functions (Zitvogel and Kroemer, 2012; Yi et al., 2021). Blocking PD-1/PD-L1 signaling has shown remarkable effectiveness in restoring T cells from an exhausted status, and normalizing the dysregulated TME, ultimately leading to cancer cell eradication (Cha et al., 2019). So far, the use of antibodies against the PD-1/PD-L1 axis has shown potent antitumor activities in several cancer types, including lung adenocarcinoma (LUAD), gastric cancer, melanoma, and liver cancer (Sui et al., 2015; Han et al., 2020). Determination of PD-L1 expression is one of the main parameters used to select patients who might benefit from this therapeutic approach and the PD-L1 positivity, resulting from an immune response-mediated PD-L1 expression, is often associated with a good response to anti-PD-1/PD-L1-based therapies. PD-L1 negative tumors, instead, are generally unresponsive to anti-PD-1/PD-L1-based treatments and only the combination with therapies promoting T-cell infiltration might be useful to improve the responsiveness to this therapeutic approach (Ribas and Hu-Lieskovan, 2016).

The understanding of the biological mechanisms underlying PD-L1 regulation is still a very hot topic in cancer biology and new insights are ever-growing. PD-L1 can be modulated by both intrinsic (i.e., cancer cell-associated) and extrinsic (i.e., originating from the TME) factors (Wu et al., 2019; Hudson et al., 2020). The intrinsic factors include dysregulation of oncogenic signaling pathways (i.e., JAK/STAT, ERK/RAS, PI3K/AKT/mTOR) which leads to the abnormal activation of specific transcription factors such as c-Myc, HIF-1α, STAT3, NF-κB, and Nrf2 (Zerdes et al., 2018). Alternatively, the expression of PD-L1 may depend on inflammatory signals, cytokines, and metabolites (i.e., IFN-α, TNF-α, IL-6) arising from the tumor cells themselves or from the TILs, the antigen-presenting cells (APC), and the tumor-associated macrophages (TAMs) (Zhang et al., 2017). To make it more puzzling, intrinsic, and extrinsic factors may regulate PD-L1 expression in multiple ways including genomic alterations, epigenetic modification, transcriptional regulation, post-transcriptional modification, and post-translational modification (Ju et al., 2020).

TME is a complex and continuously evolving entity (Anderson and Simon, 2020). Its features vary between tumor types as a consequence of a complex interplay between tumor cells, non-tumor cells and non-cellular components such as nutrients and extracellular matrix proteins. Recent evidence highlights that tumor and non-tumor cell populations within the TME dynamically communicate with each other through metabolic connections, causing a reciprocal metabolic interplay. Such metabolic symbiosis not only reprograms both anabolic and catabolic processes in the recipient subpopulations but also rewrites cancer mass evolution (Comito et al., 2020). As such, TME is now considered of a complex ecosystem that supports tumor growth, progression, and metastatic dissemination and therefore a promising target for therapy (Pitt et al., 2016; Xiao and Yu, 2021).

Iron is a multifunctional micronutrient involved in different signaling pathways within tumor cells as well as between tumor cells and the surrounding TME (Sacco et al., 2021). Iron can favor cancer progression by acting as a cofactor for enzymes involved in ATP production, DNA replication, and repair (Brown et al., 2020). For these reasons, tumor cells tend to exhibit an “iron-addicted” phenotype. To satisfy the pronounced iron demand, cancer cells adopt two different strategies. The first one is the reprogramming of the intracellular iron metabolism, through the overexpression of proteins involved in iron uptake (i.e., transferrin receptor, CD71) and storage (i.e., ferritin heavy subunit, FtH) and the parallel downregulation of proteins involved in iron export (i.e., ferroportin, FPN) (Zolea et al., 2016; 2017; Pfeifhofer-Obermair et al., 2018; Chirillo et al., 2020). The other one is the diversion of tumor-infiltrating immune cells, in particular tumor-associated macrophages (TAMs) and neutrophils residing in the TME, which can either serve as sources of iron and iron-related proteins or release factors that activate signaling pathways involved in the control of iron metabolism in cancer cells (Sacco et al., 2021). In parallel, functional, metabolic, and immunological features of the TME rely on major shifts in iron metabolism. Indeed, M2-polarized TAMs, showing an “iron-releasing” phenotype, appear to sustain cancer growth through the capacity to repress anti-tumor immune functions. (Cassim and Pouyssegur, 2019). From their side, cancer cells with a pronounced iron demand may deprive the TME of iron, thus producing and releasing ROS, which in turn may suppress the antitumor activities of immune cells (Ying et al., 2021).

The iron addiction phenotype is, however, a double-edged sword as the accumulation of the labile and redox-active iron pool (LIP) within the cytoplasm may lead to the generation of reactive oxygen species (ROS), which in turn cause oxidative damage and eventually ferroptosis (Battaglia et al., 2020; 2022). In this regard, it has been recently reported that “iron-retaining” M1 TAMs, showing a pro-inflammatory M1-like phenotype, induce tumor cell death and through the generation of ROS and pro-inflammatory cytokines (TNFα and IL-6) (Sacco et al., 2021). Costa da Silva M et al. have demonstrated that, when exposed to distinct iron sources such as hemolytic red blood cells, TAMs polarize towards the M1 pro-inflammatory phenotype and exert a marked anti-tumor activity in LUAD. Notably, this effect is also elicited by the intra-tumoral injection of iron oxide nanoparticles, which significantly reduce tumor size *in vivo* (da Silva et al., 2017). In hyper-inflamed tumors, the TME is over-enriched in iron, which, in turn, promotes T cell dysfunction in a ferroptosis-dependent manner (DeRosa and Leftin, 2021; Sacco et al., 2021). Data supporting the possible implication of iron and/or ROS within the TME in the regulation of immune checkpoints in cancer are still very poor. In breast cancer, treatment with ROS inducers (i.e., paclitaxel or buthionine sulphoximine) promotes the transcription of PD-L1 via NF-κB in TAMs, which in turn acquire an immunosuppressive phenotype and improve the efficacy of anti-PD-L1 antibody-based immunotherapy (Roux et al., 2019). A very recent study conducted on C57Bl/6N female mice with implanted E0771 mammary carcinoma cells, highlights that *in vivo* iron supplementation increases the availability of this metal in the TME and that this is accompanied by suppression of T cells activation as well as by the reduction of anti-PD-L1-based therapy efficacy (Tymoszuk et al., 2020). Recently, Choi EJ et al. demonstrated that ferric ammonium citrate (FAC) induces PD-L1 in bone marrow-derived macrophages (BMDMs macrophages) and that this is mediated by its redox activity (Choi et al., 2022).

In this study, we show for the first time that a high iron density within the TME is associated with high PD-L1 expression levels in LUAD tissue specimens. Furthermore, we demonstrate that *in vitro* supplementation of iron within culture media induces PD-L1 overexpression through the generation of ROS which, in turn, activates the PD-L1 transcription factor c-Myc. The iron-mediated PD-L1 overexpression in LUAD cells inhibits T-cell activation in co-culture conditions.

## 2 Materials and methods

### 2.1 Patients and tissue samples

Sixteen archived LUAD samples (formalin-fixed, paraffin-embedded (FFPE) blocks) were kindly provided by the Annunziata Hospital (Cosenza, Italy). Samples were collected during the surgical tumor resection between 9 January 2020, and 10 November 2020. Informed consent was obtained from all subjects. The main clinical information associated with each sample were not correlated with any clinical studies or immune checkpoint inhibitor therapy.

### 2.2 PD-L1 immunohistochemical staining

The immunohistochemical (IHC) staining for PD-L1 was performed by using the FDA-approved Dako Agilent PD-L1 IHC 22C3 pharmDx kit on the Dako Autostainer Link 48 platform. PD-L1 positive control material for protocol establishment included FFPE specimens from normal tonsil. The analysis was limited to cell blocks with >100 tumor cell. The analysis of stained tissue samples was performed by 2 expert pathologists involved in the routine evaluation of clinical samples for diagnostic purposes. The estimation of PD-L1 expression below or beyond the 2 cut-off points (1% or 50%) were done according to current clinical practice for the determination of patients eligible for anti-PD-1 treatments. According to the Tumor Proportion Score (TPS) patients were classified as low TPS (1%–49%) and high (TPS ≥50%) expressors, respectively (De Marchi et al., 2021).

### 2.3 Perls Van-Gieson iron staining

To determine the iron content in both intra-tumoral and peritumoral TAMs, each tumor sample was stained by using Perls Van-Gieson Kit (Bio-Optica). Briefly, paraffin-embedded tissue samples were rehydrated, stained with Perls solution for 20 min and then rinsed in distilled water. Sections were then counterstained with Van-Gieson solution for 10 min and, rinsed in water. Finally, sections were dehydrated in 70%, 95%, 100% ethanol and embedded using xylene-based mounting media. Producer guidelines have been used to quantify the positivity or negativity to Perls staining. Patients were defined as iron positive or negative according to the positivity (despite of the threshold) or negativity to Perls staining.

### 2.4 Cell lines and cell culture

LUAD cell lines A549 and H460 were purchased from the American Type Culture Collection (ATCC, Rockville, MD, United States) and grown in RPMI-1640 (Sigma-Aldrich, St. Louis, MO, United States) supplemented with (v/v) fetal bovine serum (FBS) (Invitrogen, San Diego, CA), 100 U/mL of penicillin, and 0.1 mg/mL of streptomycin (Sigma-Aldrich, St. Louis, MO, United States). Cells were maintained in a 5% CO_2_ humidified atmosphere at 37°C and periodically tested for the presence of *mycoplasma*. For each experiment, cells were seeded to obtain 70%–80% confluence.

### 2.5 Reagents

Ferlixit (62.5 mg/5 mL, SANOFI) was obtained from the outpatient pharmacy at Unit of Cardiology, Magna Graecia University, Germaneto, while the antioxidant (±)-6-hydroxy- 2,5,7,8-tetra-methylchromane-2-carboxylic acid (trolox) was ordered from Cayman Chemical (Cayman Chemical Company, Ann Arbor, United States). Cells were seeded in a 6-well plate in serum-free medium. Ferlixit was added into the medium at the final concentration of 250 µM for 24 h while trolox was used at 200 μM for 6 h.

### 2.6 Western blot

To obtain total protein extracts, protein extraction was performed using RIPA buffer as previously described (Biamonte et al., 2015; Zolea et al., 2015; 2016; Di Sanzo et al., 2022), supplemented with cOmplete™ Protease Inhibitor Cocktail provided in EASYpacks (Roche Diagnostics, Mannheim, Germany). Otherwise, nuclear, and cytoplasmic protein extracts were obtained as previously described (Chirillo et al., 2020; Scaramuzzino et al., 2021). Equal amounts of protein (50 μg) from each sample were separated by 8%–12% SDS-PAGE. The migration was performed at 200 V for 1 h and 30’. Then, proteins were transferred to nitrocellulose membranes (Sigma-Aldrich, St. Louis, MO, United States) at 50 V for 2 h. The membranes were blocked in 5% milk or 5% BSA for 1 h at room temperature and incubated overnight at 4°C with primary antibodies against PD-L1 (1:1,000, PA5-28115, ThermoFisher Scientific), c-Myc (1:500, sc-42, Santa Cruz Biotechnology), Nrf2 (1:200, sc-365949, Santa Cruz Biotechnology), NF-κB (1:500, sc-8008, Santa Cruz Biotechnology), HIF-1α (1:500, sc-10790, Santa Cruz Biotechnology) p-STAT3 (1:500, 4,113s, Cell Signaling Technology), STAT3 (1:500, 9,139s, Cell Signaling Technology), p-mTOR (1:500, 5,536s, Cell Signaling Technology), mTOR (1:500, 2,972s, Cell Signaling Technology), p-ERK (1:500, 9,106s, Cell Signaling Technology), ERK (1:500, 9,107s, Cell Signaling Technology), c-JUN (1:500, sc-1694, Santa Cruz Biotechnology), p70 S6 Kinase (S6, 1:500, 2,708, Cell Signaling Technology) and Phospho-p70 S6 Kinase (p-S6, 1:500, 9,206, Cell Signaling Technology). The membranes were washed for 30 min and then incubated for 1 h at room temperature with peroxidase-conjugated secondary antibodies (Peroxidase AffiniPure Sheep Anti-Mouse IgG, 1:10,000; Peroxidase AffiniPure Donkey Anti-Rabbit IgG, 1:10,000; Peroxidase AffiniPure Donkey Anti-Goat IgG, 1:10,000; Jackson ImmunoResearch Europe Ltd). Signals were detected using chemiluminescence reagents (ECL Western blotting detection system, Santa Cruz Biotechnology, Dallas, Texas) and acquired by Uvitec Alliance Mini HD9 (Uvitec Cambridge, United Kingdom). To calculate the relative expression of specific protein, a goat polyclonal anti-γ-Tubulin antibody (γ-TUB, 1:3,000; sc-17787; Santa Cruz Biotechnology) serves as a reference for citosolic sample loading, while Lamin A (LAMIN, 1:2000, sc-20680, Santa Cruz Biotechnology) was used as loading control for nuclear samples. The protein band intensity on western blots was quantified and normalized to that of γ-TUB or LAMIN by using ImageJ software (http://rsb.info.nih.gov/ij/).

### 2.7 Measurement of the labile iron pool (LIP)

Intracellular labile iron concentration was determined by flow cytometry using the fluorescent iron sensor calcein acetoxymethyl ester (CA-AM). Briefly, cells were incubated with 0.25 μM CA-AM (Aldrich, Missouri, United States) for 30 min at 37°C in the dark. Then, cells were washed twice with PBS (1X) to remove the excess of CA-AM, and thus treated with 200 μM L1 (3-Hydroxy-1,2-dimethyl-4(1H)-pyridone, Sigma-Aldrich, Missouri, United States) or left untreated. The analysis was performed by FACS BD LSRFortessaTM X-20 cytofluorometer (BD Biosciences). The difference in cellular fluorescence after and before incubation with L1 reflected the labile iron pool:

$$\Delta Mean\;Fluorescence\;Intensity,\Delta MFI={\Delta MFI}^{after}-{\Delta MFI}^{before}$$

### 2.8 Mitochondrial ROS analysis

Generation of mitochondrial ROS was measured by flow cytometry with the use of MitoSOX Red Mitochondrial Superoxide Indicator (Thermo Fisher Scientific Inc.). After treatments, cells were incubated with 5 µM MitoSOX Red for 10 min at 37°C, washed in PBS (1X), and then analyzed by flow cytometry using a FACS BD LSRFortessaTM X-20 cytofluorometer (BD Biosciences). A minimum of 20.000 cells was analyzed per condition. Fluorescence was measured using FlowJo software program (Tree Star, Inc.). Each experiment was performed in triplicate.

### 2.9 Flow cytometry analysis of cell surface PD-L1

For the flow cytometry analysis of surface PD-L1, cells were incubated with PD-L1 antibody (anti-human CD274, APC, BioLegend, San Diego, California, United States) for 30 min in the dark. After washing twice with PBS (1X), cells were acquired in a FACS BD LSRFortessaTM X-20 cytofluorometer (BD Biosciences). Data were analyzed using FlowJo software (Tree Star, Inc.). Three independent experiments were conducted.

### 2.10 Apoptosis analysis

For identifying cells actively undergoing apoptosis, a double staining with Annexin V and PI was performed using Alexa Fluor^®^488 Annexin V/Dead Cell Apoptosis Kit (Thermo Fisher Scientific, Waltham, Massachusetts, United States) as previously described (Scicchitano et al., 2023). After staining, cells were incubated at room temperature for 15 min in the dark. Each tube was diluted with 400 μL of Annexin Binding Buffer and then cells were analyzed by flow cytometry using the FACS BD LSRFortessaTM X-20 cytofluorometer (BD Biosciences). Data were analyzed using FlowJo software (Tree Star, Inc.). Three independent experiments were conducted.

### 2.11 Real-time quantitative reverse transcription (qRT)-PCR

Total RNA was extracted using the Trizol method (Life Technologies, Carlsbad, CA, United States) as previously described (Biamonte et al., 2017; De Vitis et al., 2023). Then, 1 µg of total RNA were retrotranscribed using High-Capacity cDNA Reverse Transcription Kit (Thermo Fisher Scientific, Waltham, Massachusetts, United States). qRT-PCR was performed using the SYBR Green qPCR Master Mix (Thermo Fisher Scientific, Waltham, Massachusetts, United States) (Guglielmelli et al., 2010; Biamonte et al., 2021). Analysis was performed on QuantStudio 3 Applied Biosystems by Thermo Fisher Scientific. The relative mRNA expression level was calculated by the 2^−ΔΔCT^ method and glyceraldehyde 3-phosphate dehydrogenase (GAPDH) and actin beta (ACTB) were used as the housekeeping genes (Di Sanzo et al., 2016; 2018).

### 2.12 c-Myc transient knock-down

A549 and H460 cells were transfected using Lipofectamine 3,000 transfection reagent (Thermo Fisher Scientific, Waltham, Massachusetts, United States) according to the manufacturers’ protocol. c-Myc transient knockdown was performed by using a specific c-Myc siRNA (s912, Thermo Fisher Scientific, Waltham, Massachusetts, United States). To ensure an optimal control, the two cell lines were further transfected with Silencer™ Select Negative Control siRNA (Thermo Fisher Scientific, Waltham, Massachusetts, United States). The transfection efficiency was evaluated by Western blot at 48 h.

### 2.13 IFN-γ ELISpot assay

Human Peripheral blood mononuclear cells (PBMCs) were isolated from sodium heparin anticoagulated whole blood drawn from healthy donors. PBMCs isolation was performed through a density gradient centrifugation method using Ficoll Histopaque (Sigma-Aldrich, St. Louis, MO, United States). Then, PBMCs were cultured in RPMI 1640 medium and stimulated with Dynabeads Human T-Activator CD3/CD28 (11131D, Thermo Fisher Scientific) for 1 h at 37°C. Stimulated and not-stimulated PBMCs were co-colture for 3 h with H460 and A549 cells, previously treated with 250 μM ferlixit (24 h). T cell responses were assessed by ELISPOT assay using the Human IFN-ɣ ELISpot Kit (856.051.005S, Diaclone) according to the manufacturer’s protocol. Briefly, the assay plate was re-hydrated by washing with 1X PBS and blocked with capture antibody overnight at 37°C. The day after, culture medium collected from the above-mentioned co-cultures was added to each well and incubated for 2 h at room temperature followed by 1 h-incubation after adding both the detection antibody and the streptavidin conjugated with alkaline phosphatase. Finally, BCIP/NBT substrate solution was added, kept for 15–25 min until a color change was noted. Plate development was stopped with a water wash and the plate was air-dried at room temperature, avoiding exposure to light. Spots were enumerated using an automated spot counter (BIOSYS Bioreader 3,000 Auto Macroscope ELIspot Plate Reader) and data were expressed as mean values of triplicate determinations (Garofalo et al., 2023).

### 2.14 Analysis of the databases

Statistical analysis was performed using R environment (R: a language and environment for statistical computing, n.d.). The expression data and relative clinical information under the project TCGA Lung Adenocarcinoma (LUAD) were downloaded from the GDC Data Portal using R package TCGAbiolinks (Mounir et al., 2019). TIMER2.0 (Li et al., 2020), a comprehensive resource platform tool, was used for the systematic analysis of immune infiltrates in TCGA-LUAD dataset. Non-parametric test was used for statistical analyses, as PDL1 and CD71 expression values did not follow a normal distribution (Shapiro-Wilk test).

### 2.15 Statistical analyses

Correlation of PD-L1 expression with iron positivity in tissue samples was tested by Fisher’s exact test analysis. For all the analyses, the unpaired, two-tailed Student’s t-test was used to test for significant differences between two experimental groups. A *p*-value <0.05 was considered statistically significant. Each experiment was performed at least three times; results are, then, presented as mean ± SD.

## 3 Results

### 3.1 Iron density within the TME correlates with PD-L1 levels in LUAD tissue samples

**FIGURE 1:**
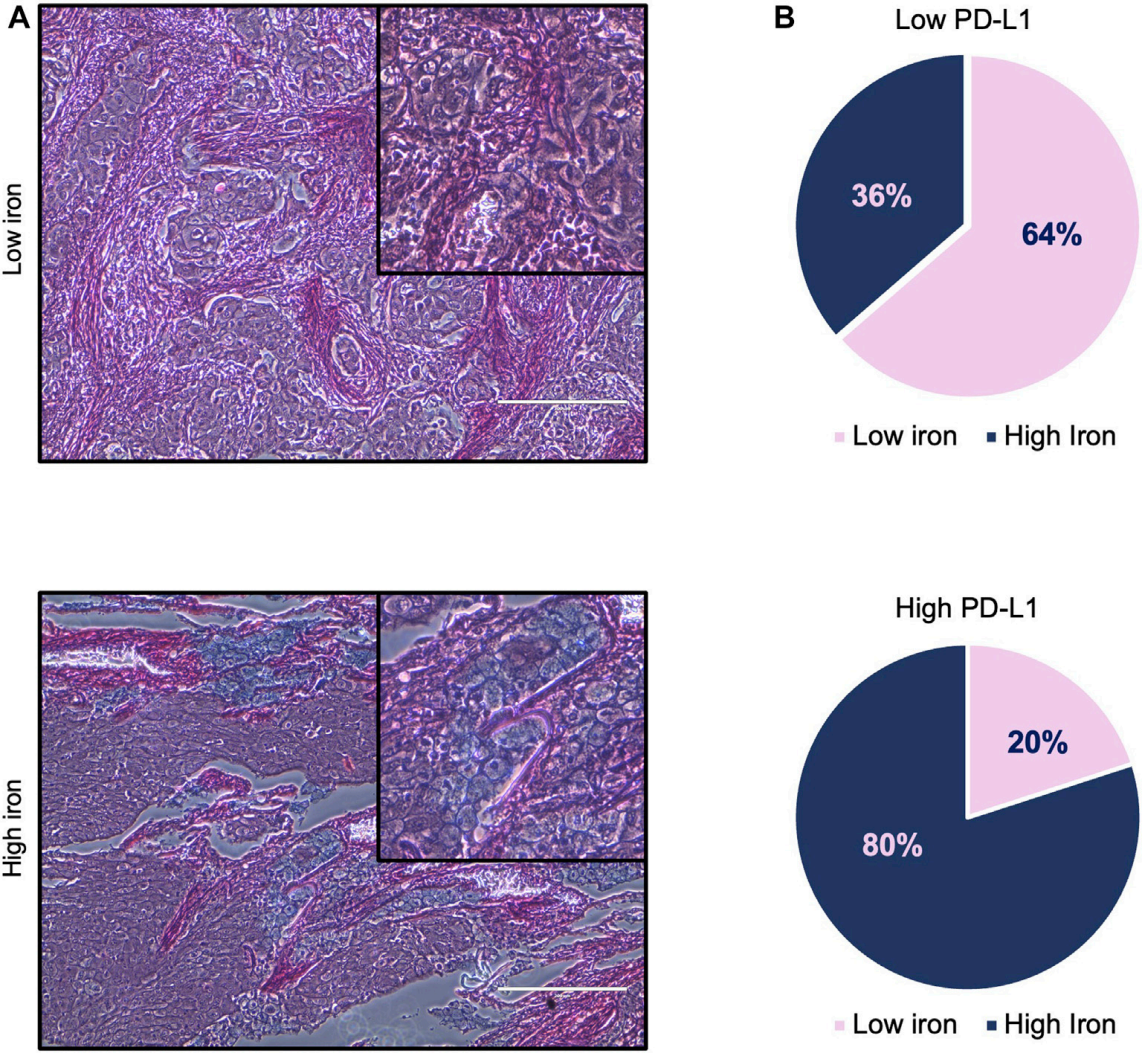
High iron density within the TME is associated with higher PD-L1 levels in LUAD tissue samples. **(A)** Representative images of LUAD tissue specimens with low (top) and high (bottom) iron content quantified by Perls Van-Gieson staining. **(B)** Pie charts representing the correlation between iron content within the TME and PD-L1 expression on tumor cells. Scale bars: 200 μm. Fisher Exact Test: *p* < 0.001.

First, we assessed whether and how PD-L1 expression in tumor cells correlates with iron content within the TME on FFPE tissue samples derived from 16 LUAD patients, whose clinicopathological features are reported in Table 1. Upon staining with the 22C3 clone anti-PD-L1 antibody, according to the TPS, 5 out of 16 samples (31%) were classified as “high PD-L1 expressors” (TPS ≥50%) while 11 out of 16 (69%) were classified as “low PD-L1 expressors” (TPS = 1–49%). By using the Perls Van-Gieson staining method, samples were also classified as “low iron” when the presence of iron inclusions within the TME were not detectable (see representative image in Figure 1A, top left) while samples with clearly recognizable iron inclusions were considered as “high iron” (Figure 1A, bottom left). Notably, 7 out of the 11 patients (64%) with low PD-L1 expression presented a low iron density within the TME, while 4 out of the 5 patients (80%) expressing high levels of PD-L1 presented a high iron density (Figure 1B, Fisher Exact Test: *p* < 0.001). These results suggest that a higher iron density within the TME might correlate with higher PD-L1 expression levels in LUAD cells.

**TABLE 1:** Clinicopathological features of 16 LUAD patients.

	Low PD-L1 expression (1%–50%)	High PD-L1 expression (>50%)	
Variables	n (%)	n (%)	*p*-value
*Sex*			n.s
Male	5 (45.5)	4 (80)	
Female	6 (54.5)	1 (20)	
*Age*			*p* < 0.001
<65 years	3 (27.3)	1 (20)	
≥65 years	8 (72.7)	4 (80)	
*Pleural invasion*			*p* < 0.001
Absent	10 (91)	4 (80)	
Present	1 (9)	1 (20)	
*Venous and lymphatic invasion*			*p* < 0.001
Absent	11 (100)	3 (60)	
Present	0 (0)	2 (40)	
*Metastasis*			*p* < 0.0001
Absent	10 (91)	5 (100)	
Present	1 (9)	0 (0)	
*Iron content*			*p* < 0.001
Low	7 (64)	1 (20)	
High	4 (36)	4 (80)	
Tot	11	5	

### 3.2 Iron addition within culture media causes oxidative stress and PD-L1 overexpression in LUAD cells

**FIGURE 2:**
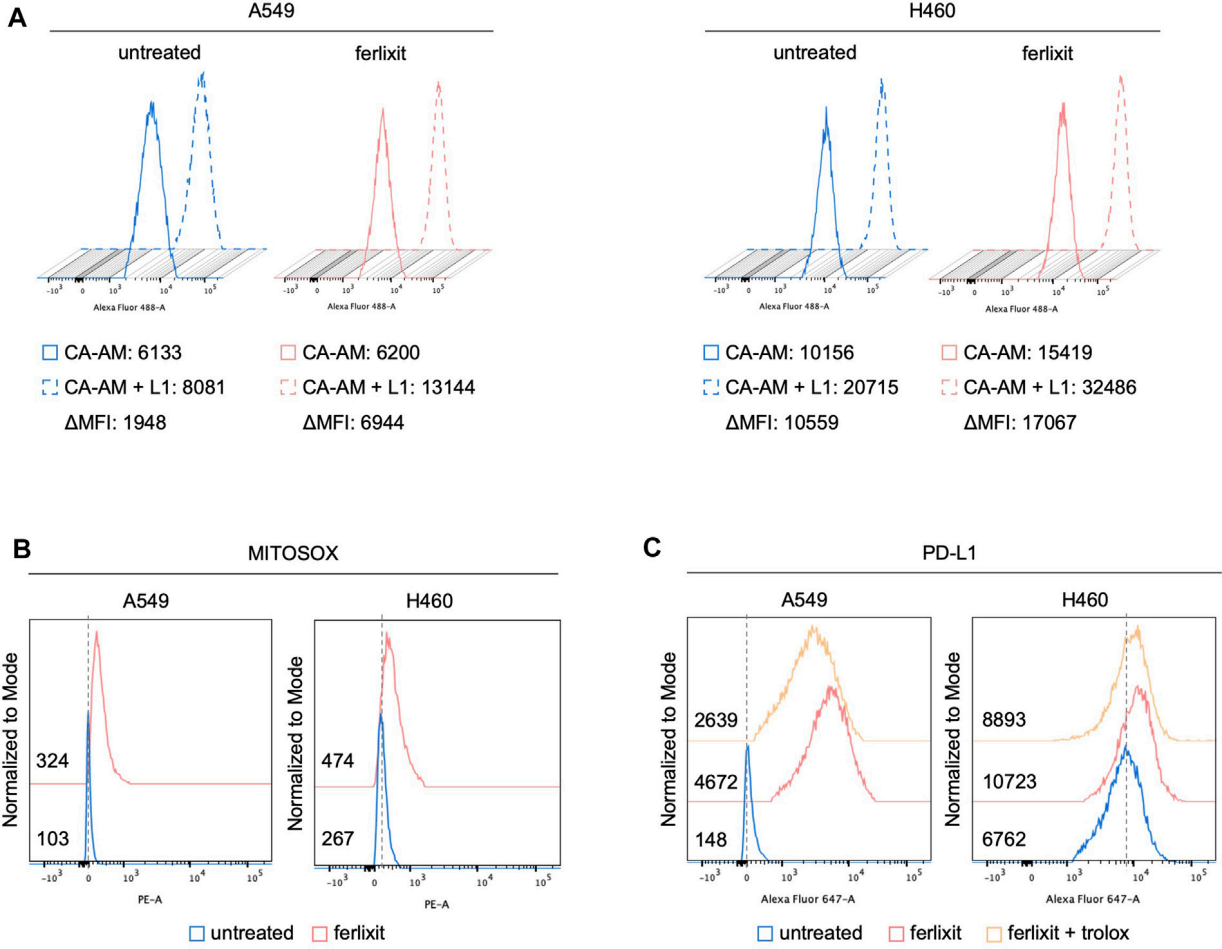
Ferlixit administration induces an increase in ROS amounts and PD-L1 overexpression in LUAD cells. Flow cytometry analysis of LIP amounts **(A)** and mitochondrial ROS levels **(B)** quantified by using CA-AM and MitoSOX reagent, respectively, in A549 and H460 cells untreated and treated with 250 μM ferlixit for 24 h. **(C)** Flow cytometry analysis of PD-L1 surface levels in A549 and H460 cells untreated or treated with 250 μM ferlixit for 24 h alone or in combination with 200 μM trolox for 6 h.

In light of the direct correlation between intratumoral iron density and PD-L1 levels, we hypothesized that iron levels could causally regulate PD-L1 expression. To this end, we investigated the effects of iron addition within culture media on PD-L1 expression in LUAD cell lines. To this, we cultured A549 and H460 for 24 h in their relative culture media with or without 250 µM ferlixit, a Fe^3+^ compound normally used to treat patients with anemia. As shown in Figure 2A, when growth in the iron-rich culture condition, H460 and A549 cells show an intracellular accumulation of free-iron (A549^untreated^ ΔMFI: 1948; A549^ferlixit^ ΔMFI: 6,944; H460^untreated^ ΔMFI: 10,559; H460^ferlixit^ ΔMFI: 17,067), and an overproduction of mitochondrial ROS (A549^untreated^ MFI: 103; A549^ferlixit^ MFI: 324; H460^untreated^ MFI: 267; H460^ferlixit^ MFI: 474, Figure 2B). This is accompanied by a significant upregulation of PD-L1 surface levels in both th cell lines (Figure 2C). The strength of the upregulation inversely correlates with PD-L1 steady state amounts of the two LUAD cell lines: in H460 cells, showing very high levels of baseline PD-L1, iron causes a 2-fold increase, while in A549, with very low baseline PD-L1 expression, iron determines more than 20-fold increase. Notably, in both cell lines, PD-L1 overexpression is consistently counteracted by treatment with the antioxidant and ROS quencher trolox (200 µM for 6 h) (A549^untreated^ MFI: 148; A549^ferlixit^ MFI: 4,672; A549^ferlixit+trolox^ MFI: 2,639; H460^untreated^ MFI: 6,762; H460^ferlixit^ MFI: 10,723, H460^ferlixit+ferlixit^ MFI:8,893, Figure 2C), thus strongly suggesting that iron promotes PD-L1 overexpression in a ROS-dependent manner. No signs of apoptosis or ferroptosis are detectable by annexin/PI flow cytometry analysis, thus excluding the possibility that PD-L1 overexpression in A549 and H460 is determined by potential cytotoxic effects triggered by the iron-mediated oxidative stress (Supplementary Figure S1).

### 3.3 Iron-mediated PD-L1 upregulation occurs at transcriptional level through c-myc activation

**FIGURE 3:**
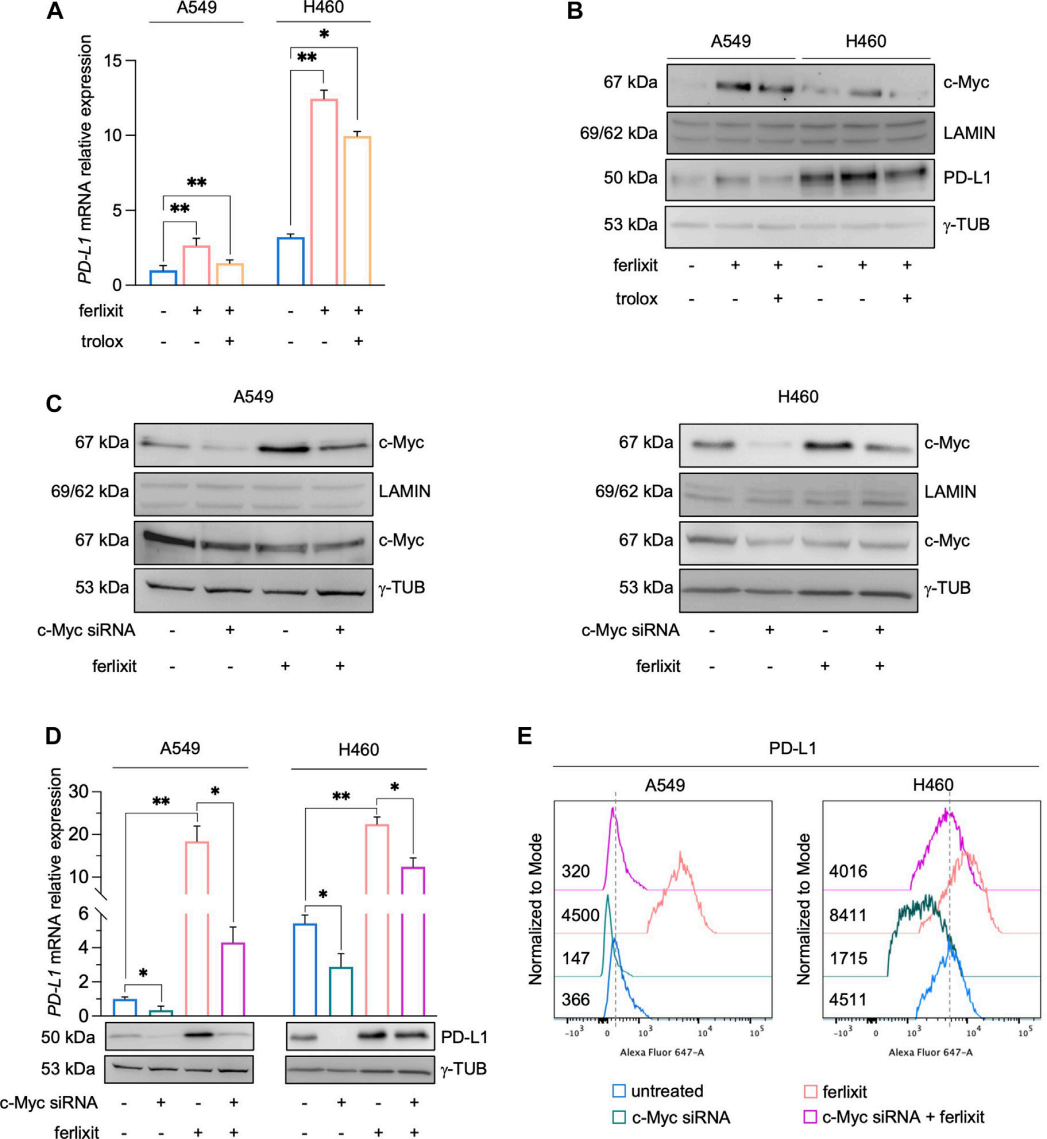
Ferlixit-dependent PD-L1 upregulation is mediated by the transcription factor c-Myc in A549 and H460 cells. **(A)** qRT-PCR of PD-L1 mRNA levels in A549 and H460 cells upon administration of 250 μM ferlixit for 24 h or additionally treated with 200 μM trolox for 6 h. **(B)** Western blot of PD-L1 and nuclear c-Myc protein levels in A549 and H460 cells upon administration of 250 μM ferlixit for 24 h or additionally treated with 200 μM trolox for 6 h. LAMIN was used as a normalization control for nuclear protein quantification, while γ-TUB as cytosolic loading control. **(C)** Western blot of PD-L1 and nuclear/cytosolic c-Myc in A549 and H460 cells untreated or treated with ferlixit (250 μM for 24 h) and upon c-Myc knockdown for 48 h. **(D)** Real-time PCR and Western Blot analyses of PD-L1 expression in A549 and H460 cells untreated or treated with ferlixit (250 μM for 24 h) and upon c-Myc silencing (48 h). **(E)** Flow cytometry analysis of PD-L1 surface levels in A549 and H460 cells untreated or treated with ferlixit (250 μM for 24 h) and upon c-Myc silencing (48 h). Results are presented as mean ± SD from three independent experiments. *p-value <0.05; **p-value <0.01.

In cancer cells, the expression of PD-L1 is intricately regulated either at transcriptional, post-transcriptional, and post-translational levels (Ribas, 2015; Ju et al., 2020). As shown by qPCR analysis in Figure 3A (see also Supplementary Figure S2A), we demonstrated that iron promotes PD-L1 overexpression essentially at mRNA levels and that this is accompanied by the enhanced nuclear translocation of c-Myc, a well-defined trancription factor of PD-L1 (Figure 3B). Once again, The administration of trolox reduces the translocation of c-Myc, which correlated with the lack of PD-L1 upregulation in both the cell lines (Figure 3B). No significant variations were observed, instead, for other PD-L1 transcription factors c-JUN, p-mTOR, HIF-1α, p-ERK, p-s6, NF-kB, p-STAT3, and Nrf2 (Supplementary Figure S2B). To better dissect the role of c-Myc on iron-mediated PD-L1 regulation, we performed the transient knockdown of c-Myc (48 h) in A549 and H460 cultured either in iron-rich or non iron-rich culture media. As shown in Figures 3C, D (see also Supplementary Figure S2C), c-Myc silencing alone causes a reduction of both cytoplasmic and nuclear c-Myc and a parallel downregulation of PD-L1 mRNA and protein levels. Besides, c-Myc knockdown in A549 and H460 grown in iron-rich culture conditons significantly attenuates its translocation and, as a consequence, the overexpression of PD-L1 induced by iron. The effects of c-Myc on iron-mediated PD-L1 regulation was further confirmed at surface levels (Figure 3E). Together, these results suggest that, in A549 and H460 LUAD cells, the iron-dependent PD-L1 upregulation is mediated by the transcription factor c-Myc.

### 3.4 Enhanced PD-L1 in LUAD cells growth in iron-rich culture media reduces the production of IFN-γ by T cells in a co-culture system

**FIGURE 4:**
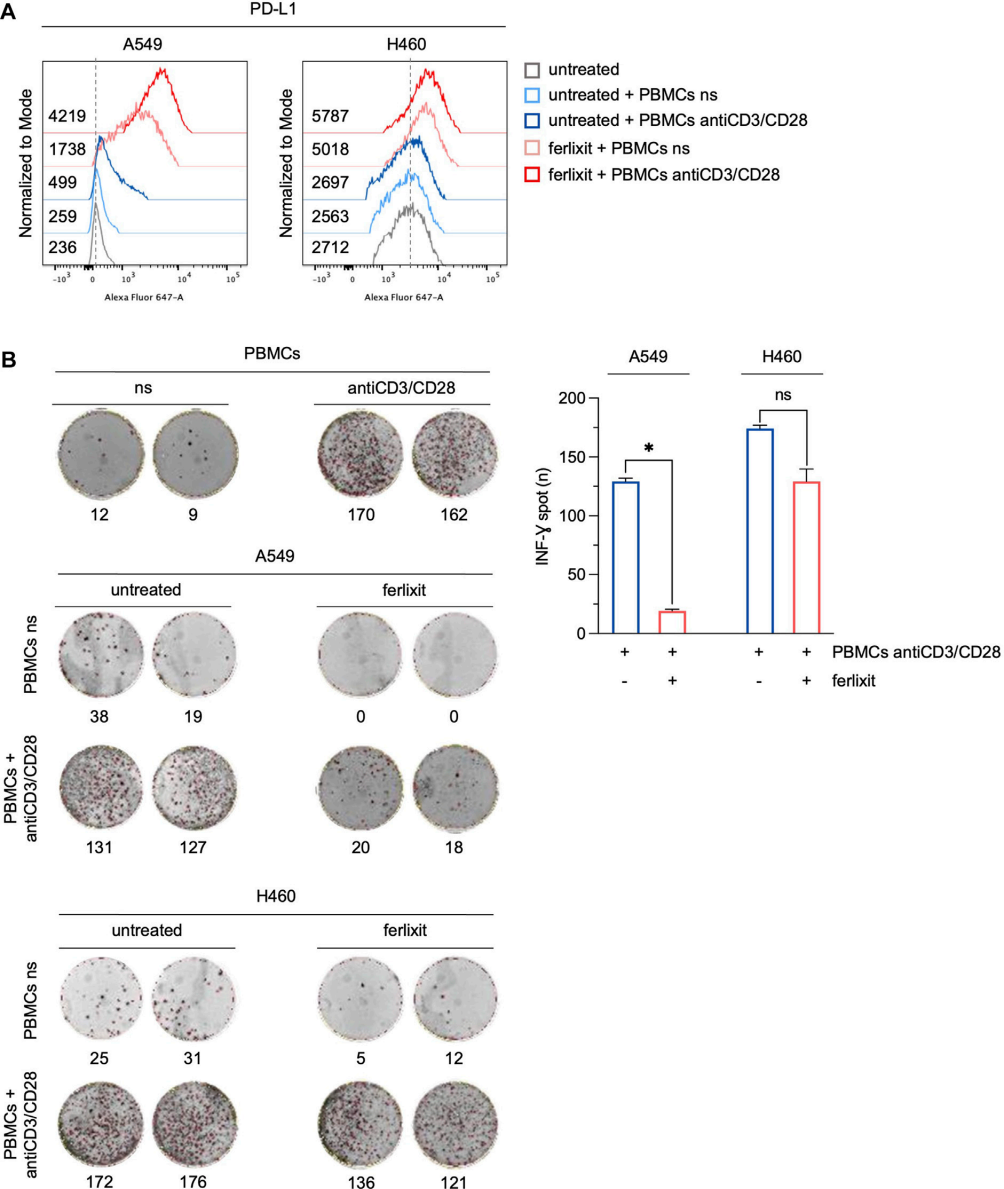
Ferlixit-induced PD-L1 overexpression reduces the release of IFN-γ by T cells in a co-culture system. **(A)** Flow cytometry analysis of PD-L1 surface levels in A549 and H460 cells treated with 250 μM ferlixit (24 h) or left untreated, and co-cultured with PBMCs either not stimulated or stimulated with antiCD3/CD28 beads. **(B)** Reactivity of PBMCs either not stimulated or stimulated with antiCD3/CD28 beads against A549 and H460 cells upon treatment with 250 μM ferlixit (24 h) in the IFN-γ ELISPOT assay. All the experiments were carried out in triplicate. The graph and error bar display data as mean ± SD. *p-value <0.05; ns: not significant.

To assess whether the overexpression of PD-L1 induced by iron in LUAD cells can affect the immune function of T cells, we measured the production and the release of IFN-γ by T cells in a co-culture system. PBMCs isolated from peripheral blood donated by healthy volunteers were stimulated with antiCD3/CD28 beads to activate T cells. Then, stimulated and not-stimulated (ns) PBMCs were co-cultured for 3 h with H460 and A549 cells in a culture medium supplemented with 250 μM ferlixit. As shown in Figure 4A, iron supplementation strongly enhances the overexpression of PD-L1 in A549 co-cultured with antiCD3/CD28 stimulated PBMCs (A549^untreated^ PD-L1 MFI: 236; A549^untreated+PBMCs ns^ PD-L1 MFI: 259; A549^untreated+PBMCs antiCD3/CD28^ PD-L1 MFI: 499; A549^ferlixit+PBMCs ns^ PD-L1 MFI: 1738; A549^ferlixit+PBMCs antiCD3/CD28^ PD-L1 MFI: 4,219). In agreement, the IFN-γ ELISPOT assay shows a remarkable reduction of IFN-γ release from stimulated PBMCs co-cultured with A549 in the iron-rich culture medium compared to those co-cultured with A549 without iron supplementation (Figure 4B). This result is partially replicated when stimulated PBMCs were co-cultured with H460 cells. Indeed, PD-L1 is only slightly overexpressed in H460 co-cultured with stimulated PBMCs in the iron-rich culture condition (H460^untreated^ PD-L1 MFI: 2,712; H460^untreated+PBMCs ns^ PD-L1 MFI: 2,563; H460^untreated+PBMCs antiCD3/CD28^ PD-L1 MFI: 2,697; H460^ferlixit+PBMCs ns^ PD-L1 MFI: 5,018; H460^ferlixit+PBMCs antiCD3/CD28^ PD-L1 MFI: 5,787) and IFN-γ release undergoes a small reduction (Figures 4A, B). Overall, these data show that an iron rich-environment promotes the overexpression of a functional PD-L1, which can significantly suppress the immune function of T cells in A549 cells. The less efficient iron-mediated induction of a functional PD-L1 in H460 cells requires further investigation; however, it can be attributed to the already very high levels of PD-L1 at baseline.

### 3.5 The expression of PD-L1 is associated with CD71 in LUAD patients

**FIGURE 5:**
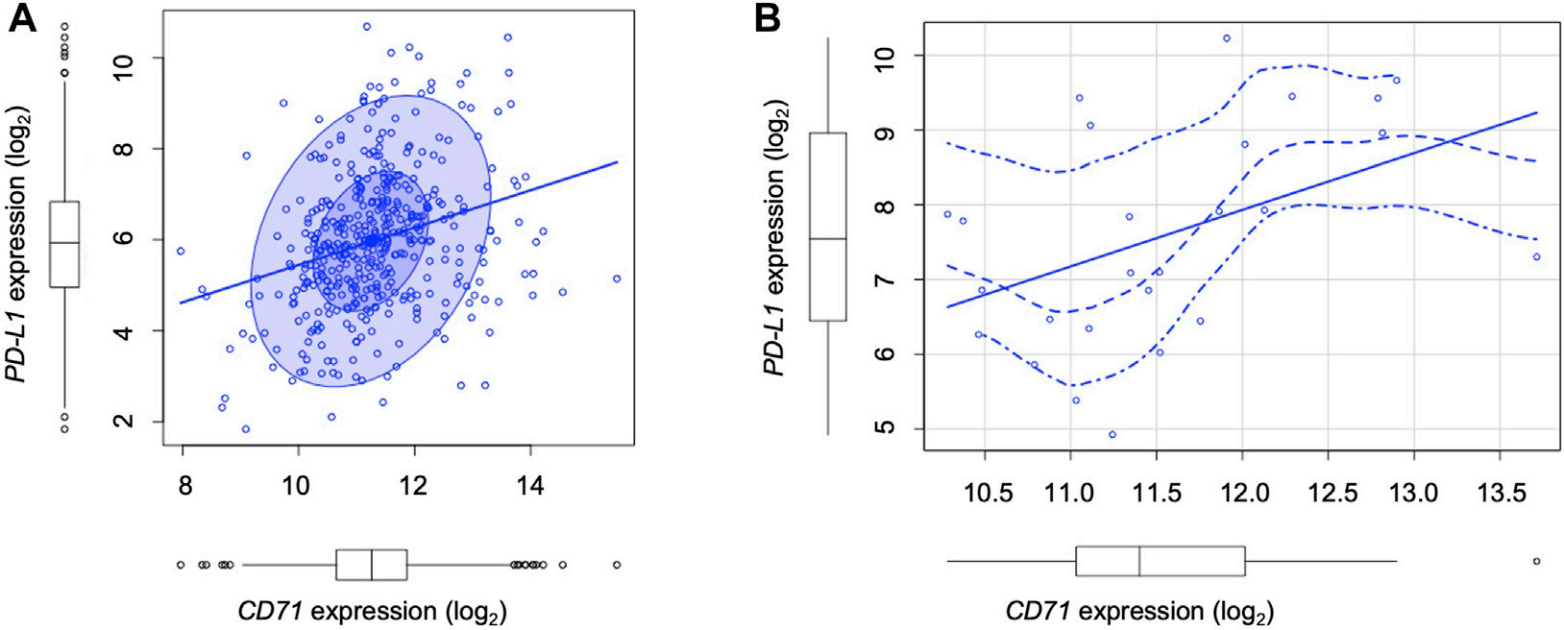
TCGA analysis show the correlation between PD-L1 and CD71 levels in LUAD patients. **(A)** Correlation between PD-L1 and CD71 mRNA levels according to the TCGA Lung Adenocarcinoma (LUAD) dataset (*n* = 433, number of patients) (*p* = 2.15e-07, rho = 0.3). **(B)** Correlation between PD-L1 and CD71 mRNA levels in LUAD patients with a higher T cell immune infiltration (*n* = 24) identified by using TIMER 2.0 (*p* < 0.004, rho = 0.6).

Cancer cells equipped with hyper functional iron uptake, often exerted by the overexpression of proteins devoted on iron intake (i.e., CD71), deprive the TME of iron to boost their protumoral functions or to suppress the anticancer activities of innate immune cells (Liang and Ferrara, 2021). Here, we finally explored whether an “iron addiction” phenotype, characterized by higher levels of CD71, may correlate with PD-L1 baseline expression in LUAD. To this, we analyzed PD-L1 and CD71 mRNA expression by using The Cancer Genome Atlas (TCGA) for LUAD dataset. We observed that in a cohort of primary LUAD patients (*n* = 433), higher levels of PD-L1 significantly correlates with higher CD71 expression levels (*p* = 2.15e-07, rho = 0.3) (Figure 5A). The correlation becomes more significant in LUAD patients with a higher T cell immune infiltration (*n* = 24) identified by using TIMER 2.0 (*p* < 0.004, rho = 0.6; Figure 5B).

## 4 Discussion

Blocking PD-1/PD-L1 axis has shown a great potential to restore TILs from exhausted status and, thus, to eradicate cancer cells (Ilcus et al., 2017). For many cancer types, the PD-1/PD-L1 status is the main speed-limiting factor of the anti-cancer immune response (Yi et al., 2021). However, a growing body of evidence suggests that oncogenic signal-mediated constitutive PD-L1, evaluated alone, could be inaccurate to select patients who might benefit from anti-PD-1/PD-L1-based therapy (Tang et al., 2015; Sui et al., 2018). Rather, understanding the difference between TME-induced PD-L1 and oncogenic signal-mediated constitutive PD-L1 can be a further valuable tool for patient selection; indeed, the environmental factors that regulate PD-L1 expression may display synergistic effects or, alternatively, elevate the sensitivity to α-PD-1/PD-L1 and other immune checkpoint inhibitors (Deng et al., 2018; Yi et al., 2021).

In this study, we aimed to investigate the impact of high iron density within the TME on PD-L1 expression in LUAD. We chose to evaluate the effects of iron on PD-L1 tumor expression in LUAD samples for two primary reasons. Firstly, although antibodies against PD-1/PD-L1 are one of the most effective immunotherapies for treating LUAD, their effects are limited to only a fraction of patients (Jiang et al., 2019). Secondly, the TME of LUAD contains one of the highest densities of TAMs (Jung et al., 2015). Our findings indicate, for the first time, that the iron density within the TME is significantly correlated with PD-L1 expression in a cohort of 16 LUAD patients. Specifically, LUAD tissue samples with higher iron density exhibit higher PD-L1 protein expression on tumor cells than those with low iron content. Based on this observation, we investigated whether iron could act as a regulatory factor for PD-L1 expression in LUAD cell lines. To do this, we treated A549 and H460 cells with ferlixit, an FDA-approved Fe^3+^ compound used to treat iron deficiency. We found that increasing the availability of iron within the culture media leads to intracellular iron overload and pronounced ROS production, resulting in a significant upregulation of PD-L1 expression. This effect is consistently counteracted by the antioxidant compound trolox, strongly suggesting that PD-L1 upregulation in LUAD cells grown in iron-rich culture conditions is ROS-dependent. Previous studies have reported that ferroptotic cancer cells overexpress immune checkpoint ligands to promote immune escape (Dang et al., 2022; Deng et al., 2022). It is important to note that the addition of iron to the culture media does not have any cytotoxic effects on A549 and H460 cells, thus excluding the possibility that PD-L1 expression is due to a phenomenon of ferroptosis.

The correlation between PD-L1 levels and tumor iron availability, which we have demonstrated experimentally, is also supported by querying the TCGA database. This publicly available resource provides gene expression and clinical information of thousands of cancer patients (Tomczak et al., 2015). Our observations in a dataset from 433 LUAD patients indicate that higher levels of PD-L1 significantly correlate with higher levels of transferrin receptor CD71, an affordable marker of cellular iron status. These findings led us to hypothesize that the pronounced iron addiction of LUAD cells, indicated by the enhanced expression of CD71, may contribute to an intrinsic propensity to overexpress constitutive PD-L1. This correlation is more significant in LUAD samples with a more pronounced T-cell infiltration, suggesting that cells with a greater ability to intake iron may release signals that foster lymphocyte recruitment within the TME. In line with this hypothesis, recent reports have shown that ferroptotic cells expose or secrete molecules such as calreticulin and HMGB, which induce the prominent activation of the immune system against tumor cells (Kroemer et al., 2013; 2022; Wiernicki et al., 2022). Therefore, ferroptosis can be classified among the “immunogenic cell-death” (Sacco et al., 2021).

The regulation of PD-L1 expression is affected by a wide array of intrinsic and extrinsic factors and is exerted either at transcriptional, post-transcriptional, and post-translational levels (Ju et al., 2020). Our results indicate that, in LUAD cells, the regulatory role of iron on PD-L1 is most likely exerted at the transcriptional level by c-Myc. The addition of ferlixit to culture media, in fact, causes c-Myc nuclear translocation and PD-L1 overexpression at mRNA levels in both H460 and A549 cells, and this effect is strongly counteracted when c-Myc is knocked down by using a specific siRNA. The role of c-Myc in regulating PD-L1 transcription has been already pointed out in many cancer types (Cha et al., 2019; Liu et al., 2022). In LUAD, Wang et al. (2017) demonstrated that overexpression of bridging integrator-1 (BIN1) reverse PD-L1-mediated immune escape by inhibiting the expression of c-Myc. Cell cycle protein-dependent kinase 7 (CDK7) inhibitor THZ1 downregulates PD-L1 expression by inhibiting c-Myc activation, and when combined with the PD-L1 inhibitor Atezolizumab improves the outcome of LUAD patients (Wang et al., 2020). Data explaining the effects of iron on c-Myc activation are, instead, still unavailable. We hypothesize that the biological mechanisms underlying c-Myc activation upon iron administration rely on the accumulation of ROS. Oxidative stress, indeed, is largely considered one of the main inducers of mitogen-activated protein kinase (MAPK) and PI3K/AKT signalling pathways, which in turn, activate c-Myc transcriptional activity through different mechanisms (Son et al., 2011). RAS signaling and the effector ERK and PI3K/AKT/GSK-3β kinase cascades induce the phosphorylation, the stabilization and thus the activation of c-Myc in melanoma cells (Tsai et al., 2012). The activation of PI3K/Akt and MAPK pathways regulates c-Myc-mediated transcription through the phosphorylation and the degradation of the c-Myc antagonist Mad1 in MCF-7 breast cancer cells (Zhu et al., 2008). At the moment, our results add iron to the list of the environmental factors able to promote PD-L1 expression and put the ground for the in-depth analysis of the biological mechanisms underlying ferlixit-mediated c-Myc activation.

In this study, we show that PD-L1 is overexpressed not only at gene but also at surface levels in A549 and H460 when grown in culture media enriched for iron, thus suggesting that environmental iron can alter tumor immune response. Indeed, we demonstrate that the release of IFN-γ by activated T cells co-cultured with LUAD cells is inhibited in culture media rich in iron, and that the inhibition of T cell activity is correlated with the levels of c-Myc/PD-L1 induced by iron. The immunosuppressive effect is particularly noteworthy in A549 cells, which exhibit a significant increase in PD-L1 expression in iron-rich culture conditions despite having low levels of PD-L1 at baseline. Conversely, H460 cells, which have high constitutive PD-L1 expression, show only a slight increase in PD-L1 expression in response to iron, resulting in little evidence of immunosuppressive activity on T cells. While we do not currently have a biological explanation for this difference, it is possible that H460 cells, like other tumor cells, use alternative strategies in addition to the PD-L1 pathway to evade immune recognition (Vinay et al., 2015). Further investigation is necessary to confirm this hypothesis. It is important to note that, as with other regulatory factors, the effects of iron on PD-L1 expression can be context-dependent, as demonstrated by previous research (Noguchi et al., 2017).

**FIGURE 6:**
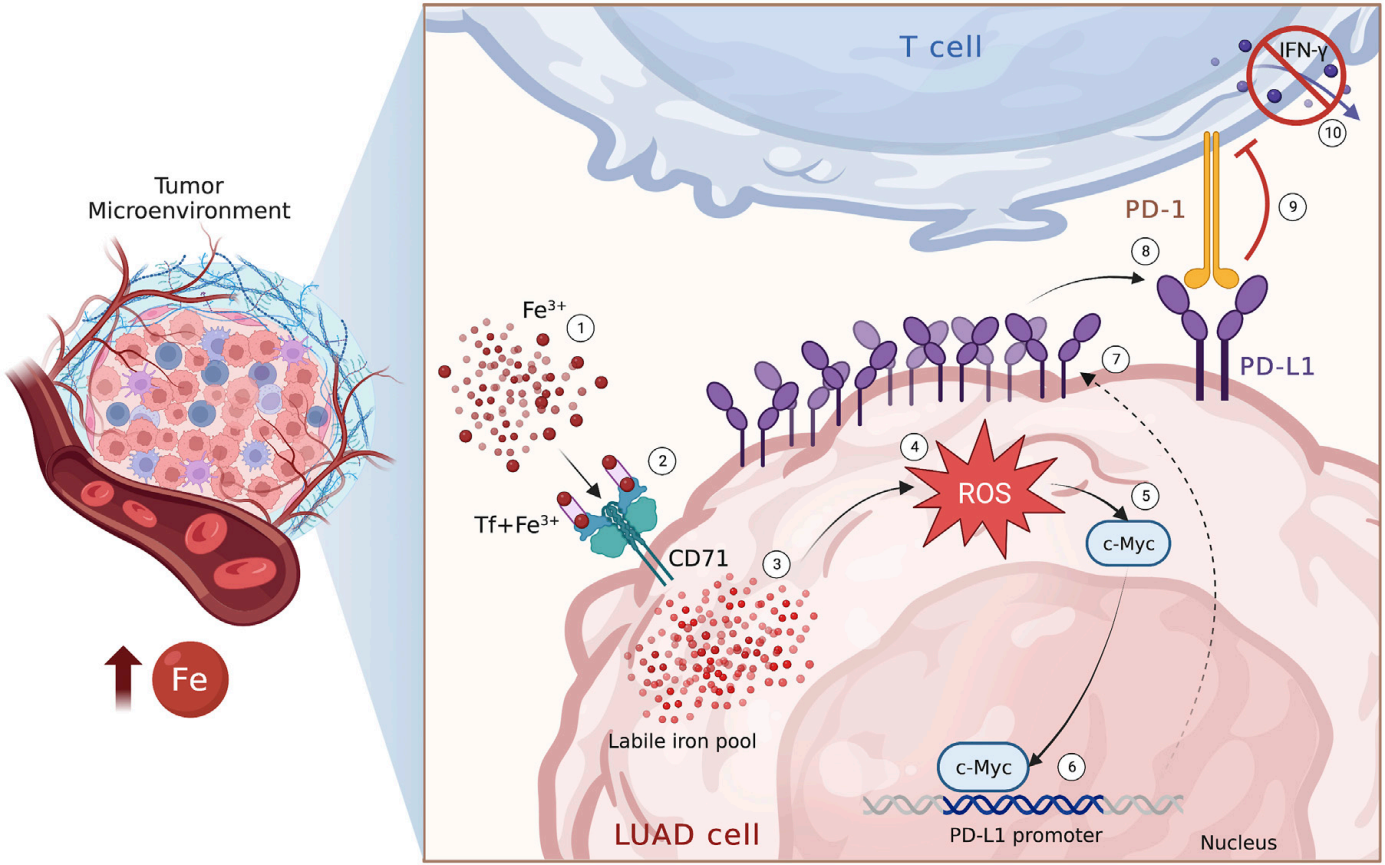
Iron within the TME promotes PD-L1 induction via ROS/c-Myc signalling pathway. Schematic representation of the iron-mediated PD-L1 induction via ROS/c-Myc signalling pathway in LUAD cells.

In conclusion, in this study we have reported, for the first time, the existence of a relationship between PD-L1 expression and iron bioavailability within the TME in LUAD samples. Additionally, we have demonstrated that iron can promote the c-Myc/PD-L1 axis in a redox-dependent manner, which may cause T cell inhibition, at least *in vitro* (Figure 6). From a clinical perspective, these findings may provide a basis for identifying and optimizing possible combinatorial strategies that consider the iron levels within the TME to enhance the therapeutic effect of immune checkpoint inhibitors and improve the outcomes of advanced LUAD patients.

## Data Availability

Publicly available datasets were analyzed in this study. This data can be found here: https://portal.gdc.cancer.gov/projects/TCGA-LUAD.
