# Supplementary material for: Iron-mediated oxidative stress induces PD-L1 expression via activation of c-Myc in lung adenocarcinoma
Source: Front Cell Dev Biol. 2023 Jun 12;11:1208485. doi: 10.3389/fcell.2023.1208485 (PMC10291098; doi:10.3389/fcell.2023.1208485)
Supplement: Supplementary file 1 [file DataSheet1.docx]

Supplementary Material

Iron-mediated oxidative stress induces PD-L1 expression via activation of c-Myc in lung adenocarcinoma

Anna Martina Battaglia^†1^, Alessandro Sacco^†1^, Ilenia Aversa^2^, Gianluca Santamaria^3^, Camillo Palmieri^2^, Cirino Botta^4^, Roberto De Stefano^5^, Maurizio Bitetto^6^, Lavinia Petriaggi^1^, Emanuele Giorgio^1^, Concetta Maria Faniello^1^, Francesco Costanzo^1,7^, Flavia Biamonte^*1,7^

***Correspondence:** Flavia Biamonte, flavia.biamonte@unicz.it

## Supplementary Figures

**Supplementary Figure 1.** Apoptotic cell death determined using Annexin V/PI flow cytometry assay in A549 and H460 cell lines upon administration of 250µM ferlixit for 24h.


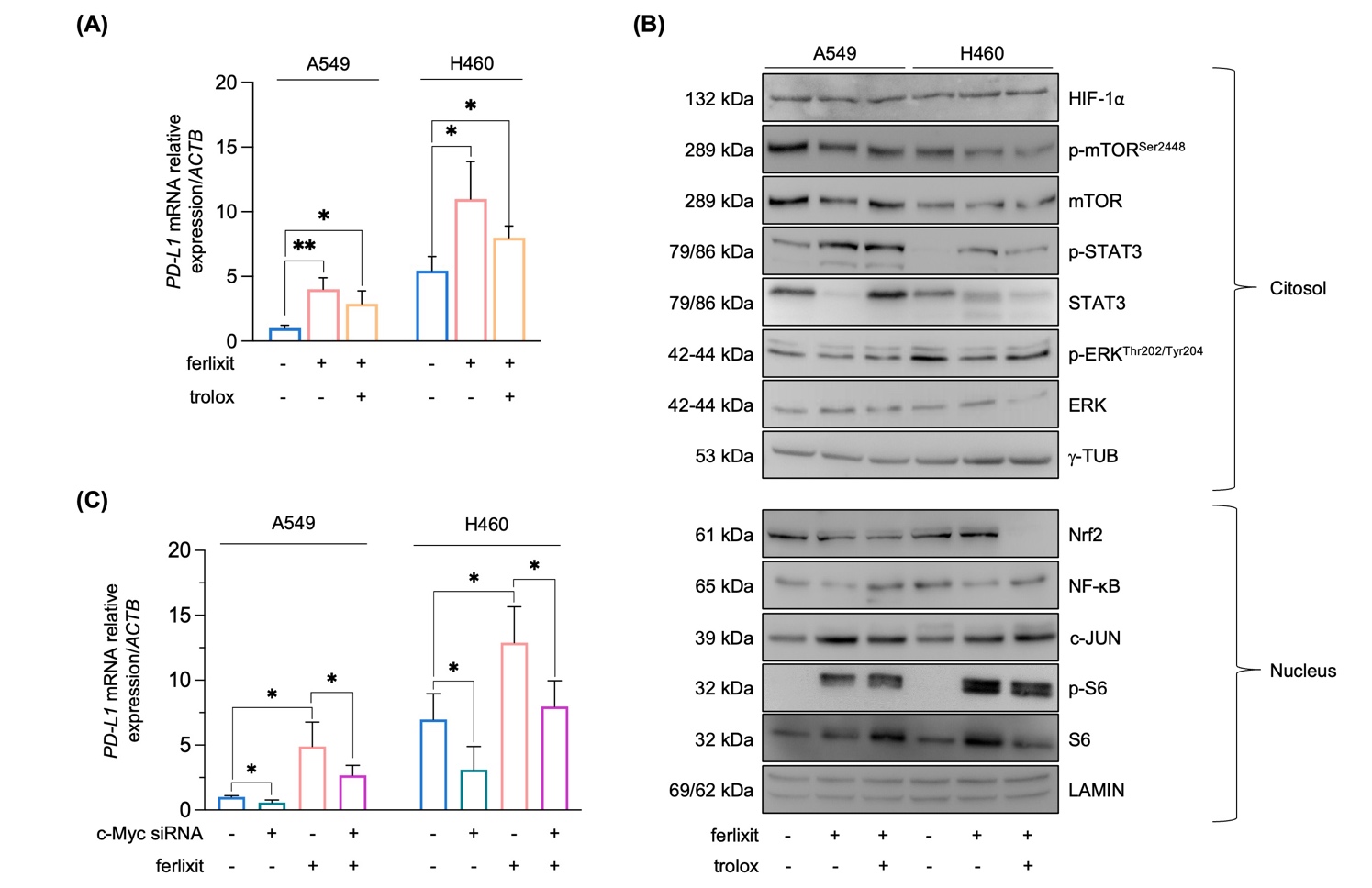


**Supplementary Figure 2.** **(A)** qRT-PCR of *PD-L1* mRNA levels in A549 and H460 cells upon administration of 250μM ferlixit for 24h or additionally treated with 200μM trolox for 6h. *ACTB* was used as housekeeping gene. **(B)** Western blot analysis of HIF-1α, p-mTOR/mTOR, p-STA3/STA3, p-ERK/ERK, NF-κB, Nrf2, c-JUN, p-S6/S6 protein expression in A549 and H460 cell lines upon administration of 250µM ferlixit for 24h alone or in combination with 200µM trolox for 6h. LAMIN and ɣ-TUB were used as a normalization control for nuclear and cytosolic protein quantification, respectively. **(C)** Real-time PCR of PD-L1 expression in A549 and H460 cells untreated or treated with ferlixit (250μM for 24h) and upon c-Myc silencing (48h). *ACTB* was used as housekeeping gene. Results are presented as mean ± SD from three independent experiments. **p*-value < 0.05; ** *p*-value < 0.01.
